# Comparative transcriptome analysis during developmental stages of direct somatic embryogenesis in *Tilia amurensis* Rupr

**DOI:** 10.1038/s41598-021-85886-z

**Published:** 2021-03-18

**Authors:** Hye-In Kang, Chae-Bin Lee, Soon-Ho Kwon, Ji-Min Park, Kyu-Suk Kang, Donghwan Shim

**Affiliations:** 1 Department of Forest Bio-Resources, National Institute of Forest Science, Suwon, 13361 Republic of Korea; 2 Department of Agriculture, Forestry and Bioresources, College of Agriculture and Life Sciences, Seoul National University, Seoul, 08826 Republic of Korea; 3 Department of Biological Sciences, Chungnam National University, Daejeon, 34134 Republic of Korea

**Keywords:** Computational biology and bioinformatics, Developmental biology, Molecular biology, Plant sciences

## Abstract

*Tilia* species are valuable woody species due to their beautiful shape and role as honey trees. Somatic embryogenesis can be an alternative method for mass propagation of *T. amurensis*. However, the molecular mechanisms of *T. amurensis* somatic embryogenesis are yet to be known. Here, we conducted comparative transcriptional analysis during somatic embryogenesis of *T. amurensis*. RNA-Seq identified 1505 differentially expressed genes, including developmental regulatory genes. Auxin related genes such as *YUC, AUX/IAA* and *ARF* and signal transduction pathway related genes including *LEA* and *SERK* were differentially regulated during somatic embryogenesis. Also, B3 domain family (*LEC2, FUS3), VAL* and *PKL,* the regulatory transcription factors, were differentially expressed by somatic embryo developmental stages. Our results could provide plausible pathway of signaling somatic embryogenesis of *T. amurensis*, and serve an important resource for further studies in direct somatic embryogenesis in woody plants.

## Introduction

*Tilia* is one of the very valuable species because they are excellent in timber materials and have beautiful tree shape in landscape. Also, there is a high demand for afforestation since they play an important role as honey trees in the summer season. But, the germination rate of *T. amurensis* is very low due to hard seed coats, immaturity of the embryo, and difficulty of penetration of moisture^1,2^. In addition, *T. amurensis* has multi-year or long-term dormancy type seedlings that take two to three years to germinate. Such low germination rate and time-consuming seedlings types, despite the high demand, make it difficult to propagate and nurture *Tilia* spp. Therefore, it is necessary to study alternative methods for mass propagation of *T. amurensis* in order to supply the seedlings to the honey farm.

Somatic embryogenesis is one of the biotechnological tools which makes somatic embryos (SE), similar in morphology to zygotic embryo. SE is bipolar structure which has both shoot apex and root apex^3^. SE can be obtained from somatic explants such as leaf, hypocotyl and zygotic embryo. SE has powerful advantages as mass propagation and allows study of morphology, physiology, and molecular mechanisms of embryo development^4,5^. Also, SE studies can provide insight into cell differentiation, totipotency, and plant regeneration^6^.

To induce somatic embryo, somatic cells must be switched to embryogenic cells which has totipotency. This process accompanies complex mechanisms such as internal, external stimuli recognition and regulatory networks^7^. Molecular mechanism of somatic embryogenesis initiation is unclear yet, but it is known that several genes are specially activated or repressed during somatic embryogenesis^8^. In order to induce SE from somatic cells, plant growth regulator (PGR) treatment is generally regarded as requisite. More than 80% of the SE induction protocol uses PGR, and most of them are auxins^9^. Exogenous auxins trigger auxin-related genes such as *YUCCA(YUC), AUXIN/INDOLE-3-ACETIC ACID (AUX/IAA), AUXIN RESPONSE FACTOR (ARF)* and cause endogenous auxin level changes^10^. Thus, hormone-responsive genes and signal transduction pathway-related genes or transcription factors that regulate hormone biosynthesis and signaling are considered to be representative regulatory genes associated with somatic embryogenesis^8,11^.

Because SE-regulatory network appears to have a high degree of complexity, overall RNA-Seq screening was adopted for transcriptome analysis. Here, we identified the genes that regulate the induction and maturation of *T. amurensis* somatic embryos through RNA-Seq screening, and described their expression patterns during somatic embryo development.

## Results

### Somatic embryogenesis for RNA-Seq analysis

**Figure 1:**
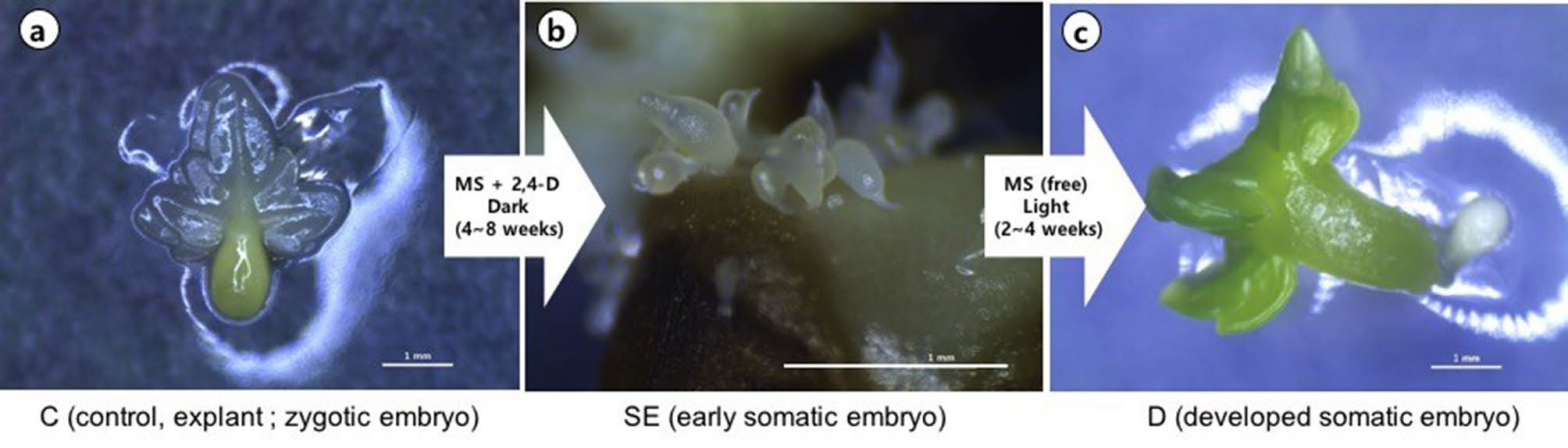
Photomicrographs of samples in stage C, SE, D. (**a**) stage C, zygotic embryo used as explant. (**b**) stage SE, directly induced early somatic embryo on the surface of explant. (**c**) stage D, matured somatic embryo. Scale bars, 1 mm. Captured by Nickon, SMZ745T.

To investigate gene expression patterns during somatic embryogenesis, we prepared tissue samples according to the developmental stages. After 8 weeks of culture, somatic embryos were directly induced from the surface of zygotic embryos. To analyze, samples were classified into three stages; C, SE, and D with three biological replicates. Stage C represents control, zygotic embryo, which is extracted from seeds and used as explant (Fig. 1a). Stage SE represents early somatic embryo which is just induced from explants (Fig. 1b). Stage D stands for matured somatic embryo, separated from SE stage and developed on medium without PGR until it looks like the control stage.

### De novo assembly of Tilia transcriptome

We conducted RNA-Seq of the C, SE and D stages with three biological replications and obtained a total of 15 × 10^6^ − 27 × 10^6^ high-quality reads per sample. In total, 192,666 transcripts and 44,350 unigenes (166,007,076 bp) were generated by Trinity assembler with high quality assembly parameters (GC contents = 42.78% and N50 = 1128 bp). After using CAP3 to merge similar genes, unigene counting reduced to 35,851 and the complete BUSCO coverage was increased from 87.4 to 87.8%.

**Figure 2:**
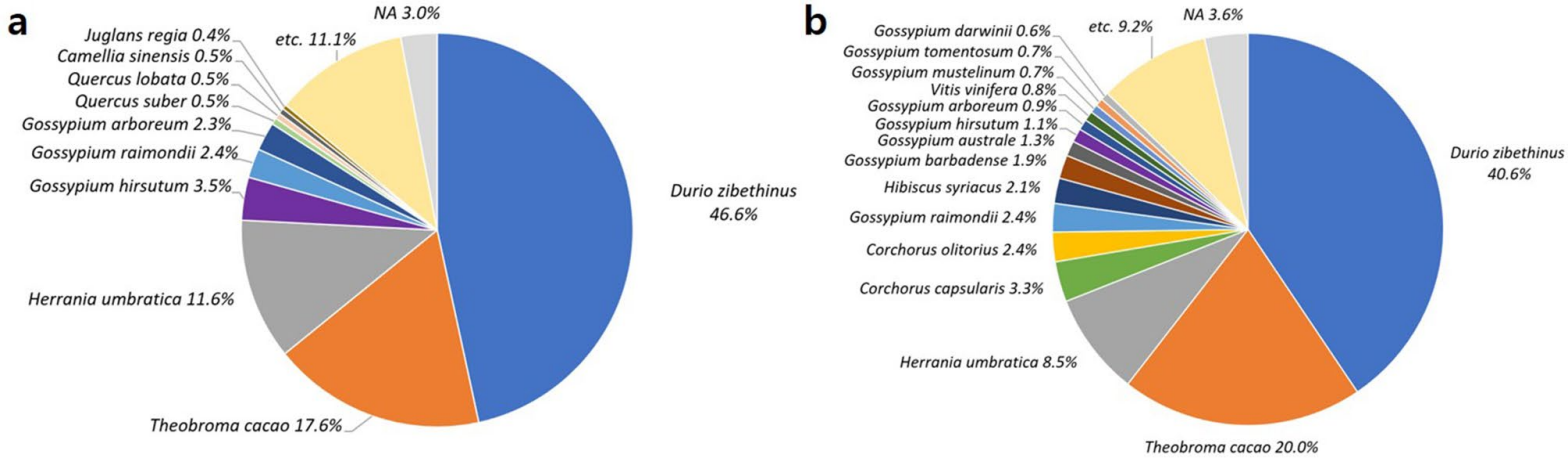
Species composition results from BLASTx of *T. amurensis* transriptome to (**a**) Refseq plant protein and (**b**) non-redundant protein databases.

Transcriptome of *T. amurensis* was most similar to *Durio zibethinus* (durian) which charges over 40% of species composition in results of BLASTx to both Refseq plant protein and non-redundant protein databases (Fig. 2). Commonly, *Theobroma cacao* (cacao), *Herrania umbratica*, and *Gossypium* species (cotton) followed Durian in species composition. These species were all included in family Malvaceae, where *Tilia* was included in.

### Identification of DEGs and GO enrichment analysis

The correlation was measured to investigate the relationship among the biological samples. Biological replicates were closely clustered in correlation heatmap (Supplementary figure S1 and S2). In total, 4592 genes were turned out to be differentially expressed genes (DEGs) in multiple comparison of C, SE and D stages with a cutoff of fold change > 4 and *p*-value < 1e^−5^. Among them, 1505 DEGs in the sub-clusters where SE stage showed distinctive expression patterns were selected and used for further DEG analysis (Supplementary figure S3).

**Figure 3:**
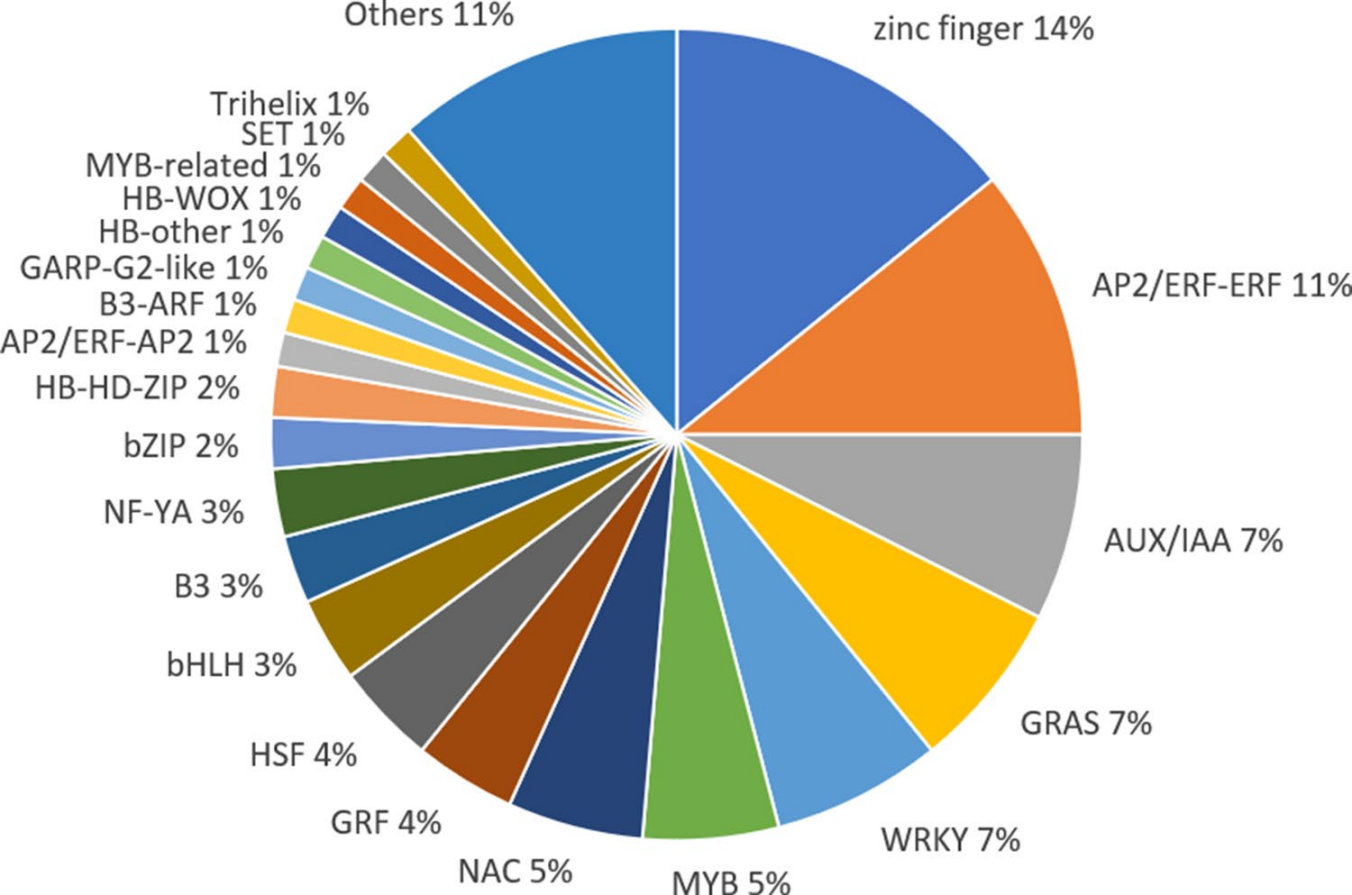
Differentially expressed TF/TR genes and classification of TF/TR families.

We annotated DEGs to Transcription Factor/Transcription Regulator (TF/TR) family identifier in order to figure out TF/TR genes present during somatic embryogenesis (Fig. 3). The proportion of TF/TR genes relative to the total DEGs was about 9.83% (148 in 1505). *ZINC-FINGER (ZNF)* and *APETALA2/ETHYLENE RESPONSE FACTOR (AP2/ERF)* family TFs occupied one quarter of all TF/TRs. The *AUX/IAA, GRAS, WRKY, MYB* and *NAC* family each counted for 5% to 7% of identified transcripts.

**Figure 4:**
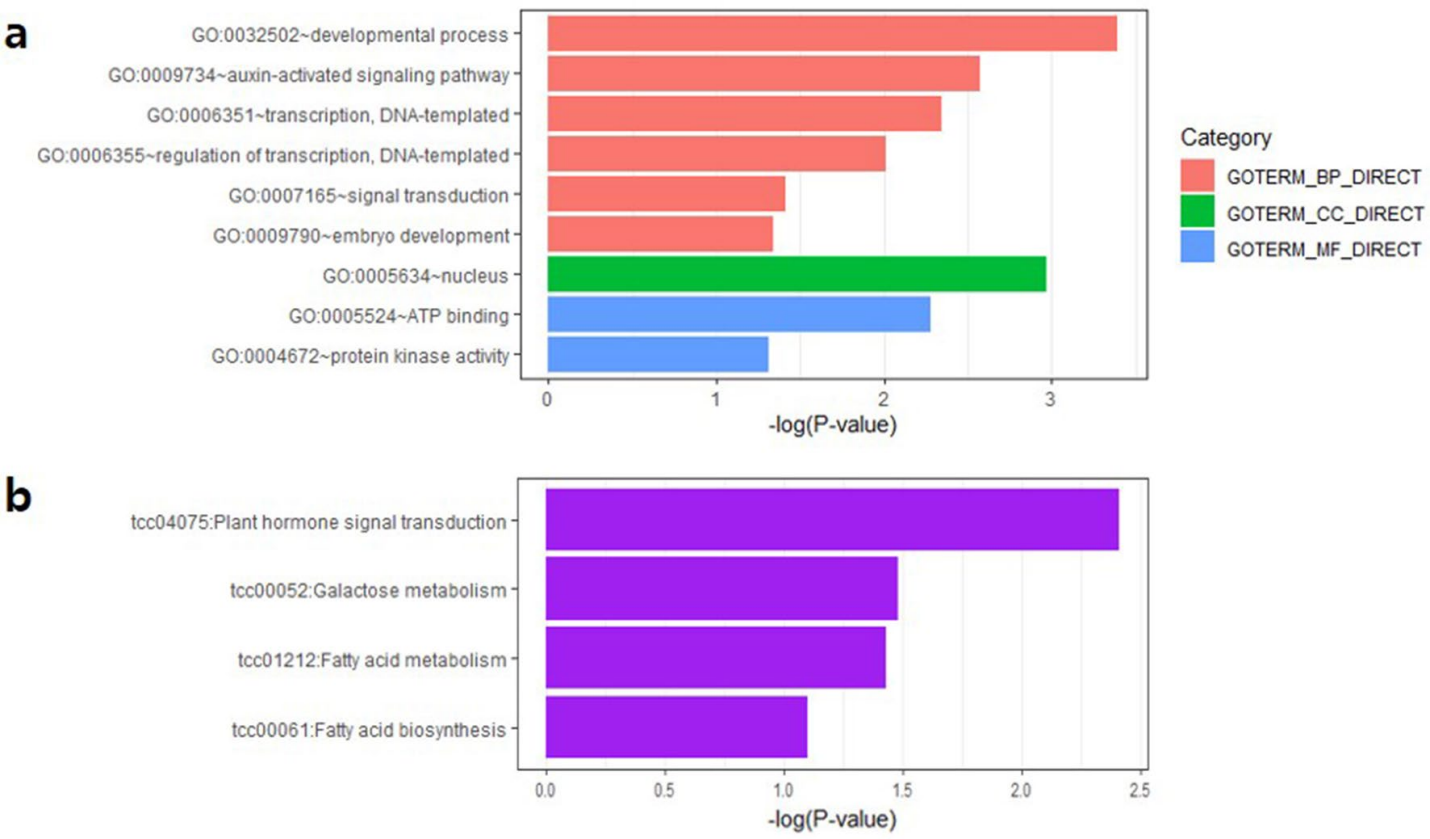
GO and KEGG enrichment analyses. (**a**) Assignment of DEGs into the GO categories (**b**) Clustering of DEGs into KEGG pathways.

The total of 75 and 18 unigenes were assigned with at least one gene ontology (GO) terms and Kyoto encyclopedia of genes and genomes (KEGG) pathways, respectively (Fig. 4, Supplementary Table 1). The DEGs were related to biological processes, cellular components and molecular functions. Developmental process, nucleus, auxin-activated signaling pathway and transcription regulation categories were significantly enriched. In addition, plant hormone signal transduction in KEGG pathway was enriched most.

**Figure 5:**
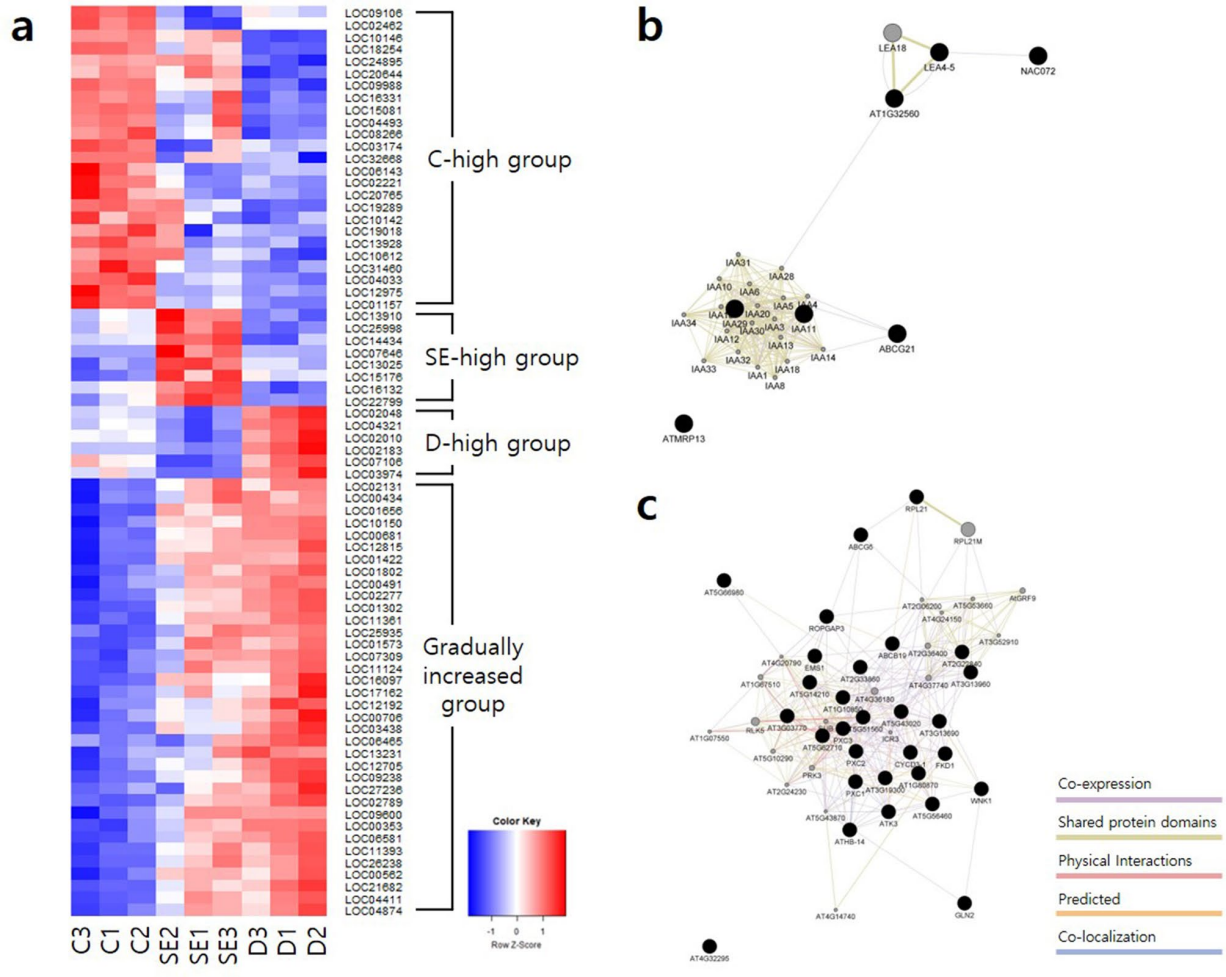
(**a**) Expression heatmap of GO assigned genes. Colors represent row-scaled TMM-normalized TPM values. (**b**, **c**) Gene functional interaction network by GeneMANIA of SE-high group and Gradually increased group. Black circle, input genes. Gray circle, neighborhood genes.

Expressions of 75 genes which were assigned with GO terms were divided into four patterns; highly expressed in C, SE, and D stage respectively and gradually increased during embryogenesis (Fig. 5a and Supplementary Table 1). SE-high group included auxin-response protein *IAA11* and *IAA20*, late embryogenesis abundant protein *D-113*, *LRR RECEPTOR-LIKE SERINE/THREONINE-PROTEIN KINASE* and *ABC TRANSPORTER*S (Supplementary Table 1). Also, gradually increased group included *LEUCINE-RICH REPEAT RECEPTOR-LIKE KINASES, CYCLIN* and *BRASSINOSTEROID INSENSITIVE 1-ASSOCIATED RECEPTOR KINASE 1 (SERK3)*. In order to define the relationship of these genes, the networks were constructed using the Cytoscape GeneMANIA app (Fig. 5b,c). Input genes and neighboring genes plugged into each other with co-expression, shared protein domain, physical interaction, predicted and co-localization relationships. The SE-high group network formation focused on the *IAA* and *LATE EMBRYOGENESIS ABUNDANT PROTEIN (LEA)* genes. Also, gradually increased group formed one large network, containing kinases such as *RECEPTOR-LIKE KINASE (RLK)* and *LEUCINE-RICH RECEPTOR-LIKE PROTEIN KINASE (PXC)*.

### Gene expression during developmtanl stages of somatic embryogenesis

**Figure 6:**
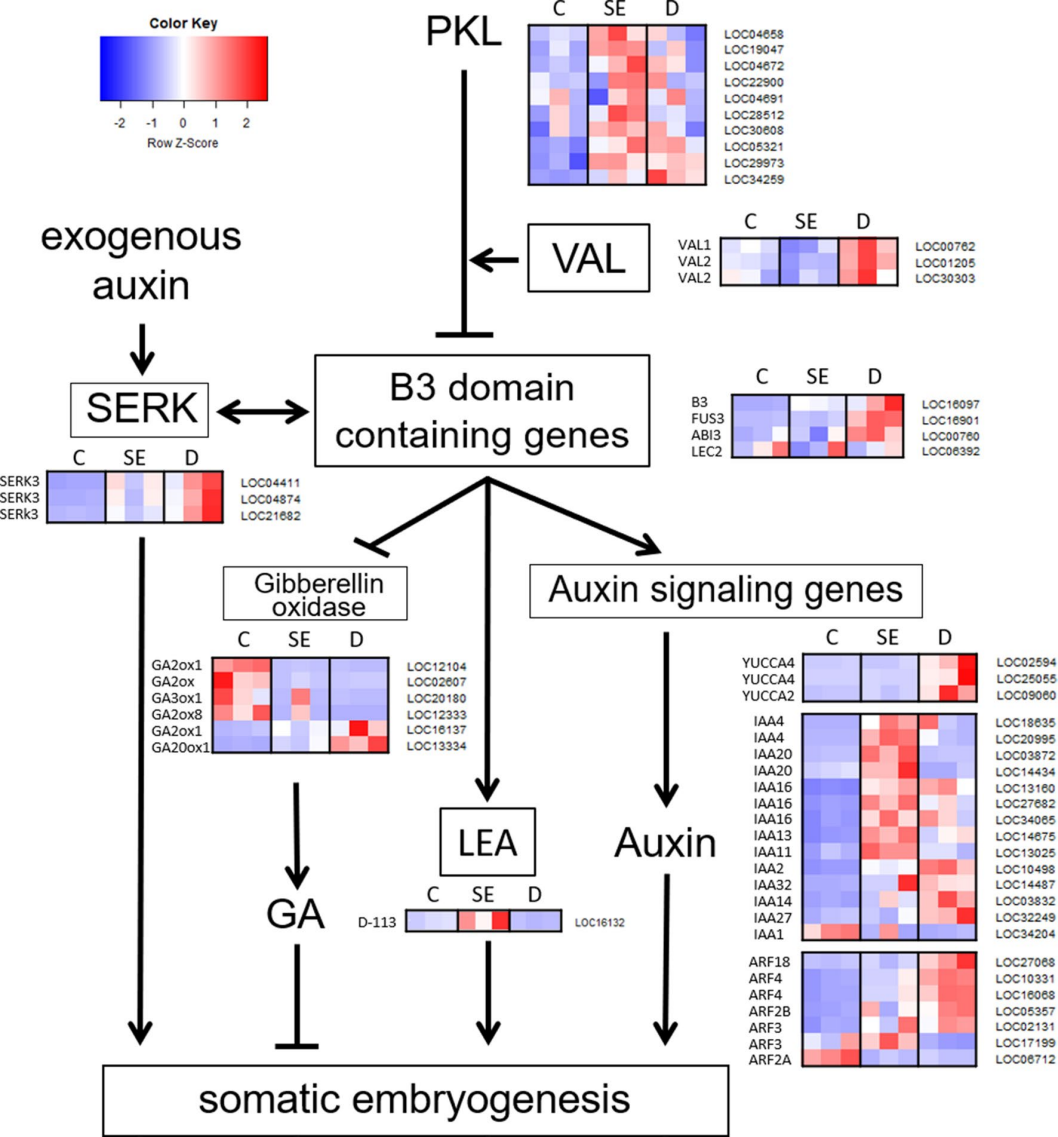
Expression level of *Tilia amurensis* genes involved in somatic embryogenesis. Colors represent row-scaled TMM-normalized TPM values.

To investigate key elements in somatic embryogenesis signaling pathways, we compared gene expression among stages (Fig. 6). Genes associated with signaling of auxin were highly expressed in SE or showed gradual increase in expression during somatic embryogenesis, whereas most of gibberellin oxidases involved in signaling of gibberellic acid (GA) were less expressed in SE and D relative to control. B3 domain containing genes that affect these phytohormone signaling and *BRASSINOSTEROID INSENSITIVE-1 ASSOCIATED RECEPTOR KINASE 1 (SERK3)* were also increased during somatic embryogenesis. Transcription factor *PICKLE (PKL)* and *VIVIPAROUS1/ABI3-LIKE (VAL)* which were known to inhibit B3 domain containing genes showed different expression patterns. *PKL* were expressed high in SE and occasionally in D, whereas *VAL1* and *VAL2* commonly showed low expression in SE and high expression in D relative to control.

### qRT-PCR conformation of expression levels of DEGs

**Figure 7:**
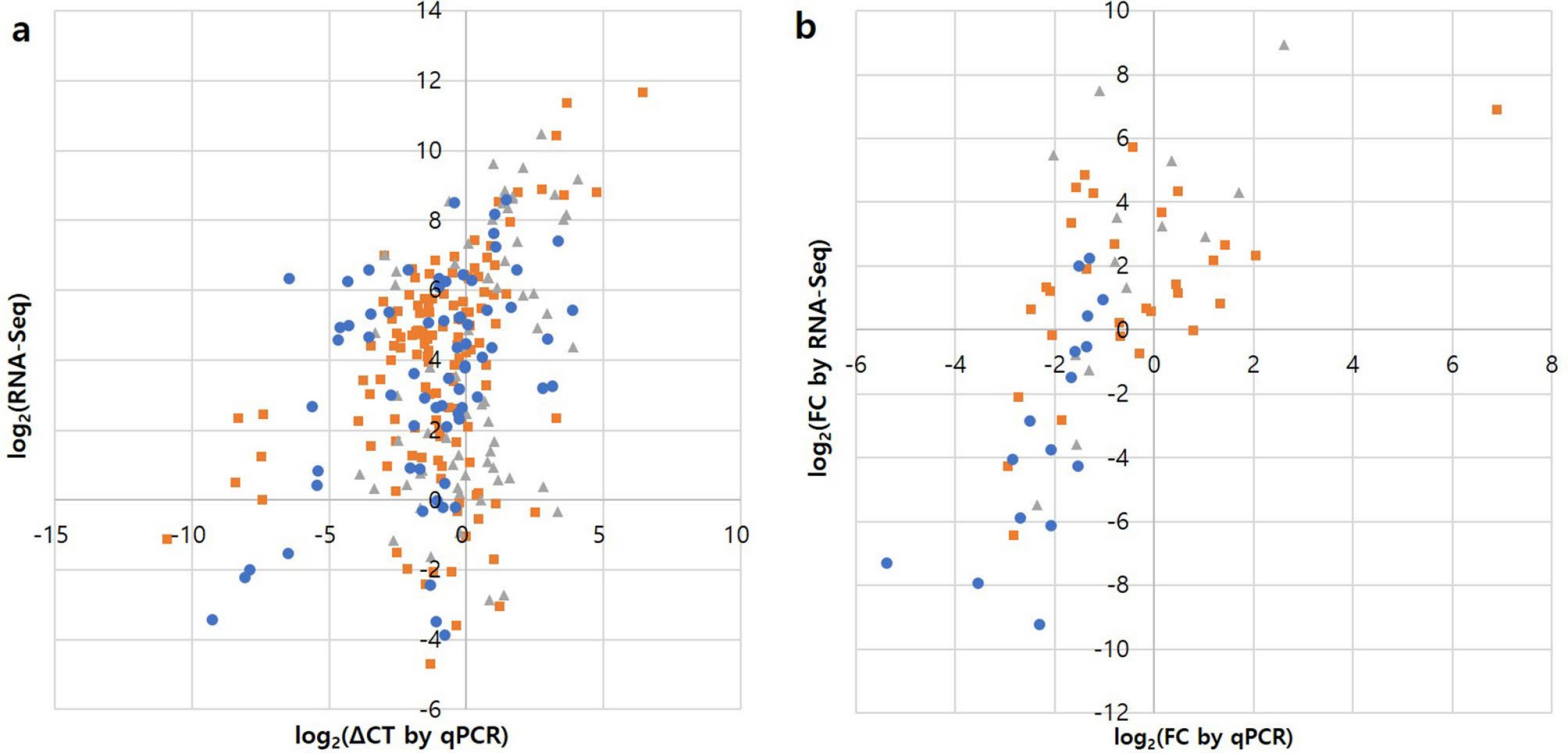
Comparison of expression profile by RNA-Seq and qRT-PCR. (**a**) Comparison between the log_2_ of qPCR ΔCT values and RNA-Seq values for every biological replicate. (**b**) Comparison between the log_2_ of gene expression ratios (versus control) from RNA-Seq data and qPCR data. Orange square, SE signaling genes; gray triangle, SE up-regulated genes; blue circle, SE down-regulated genes.

To validate RNA-Seq results, we carried out qRT-PCR for fifteen genes from Fig. 6, eight genes that showed high expression in SE samples and eight genes that showed low expression in SE samples. The qRT-PCR measurements showed moderate correlation with the RNA-Seq results when values of all samples were used in analysis individually (r = 0.38, *p*-value < 0.001, Fig. 7a). Correlation between fold change value of SE and D stage versus control by qPCR and RNA-Seq was much higher (r = 0.65, *p*-value < 0.001, Fig. 7b). That indicated RNA-Seq results was reliable. Comparison of the expression change patterns for each gene in qPCR and RNA-Seq also supported this (Supplementary figure S4).

## Discussion

*Tilia amurensis* is one of the honey sources and ornamental tree species in South Korea. The somatic embryogenesis, the way of rapid and efficient propagation for woody plant, is required in *T. amurensis* because of its inefficient reproduction manner. Previous researches on somatic embryogenesis of *T. amurensis* were mainly focused on condition for induction or morphological change^12,13^, but rarely on the molecular process. It is needed to understand molecular mechanism of somatic embryogenesis for improvement of propagation protocols. So far, the large unsequenced genome and heterozygosity have limited functional genomic analyses in *T. amurensis*. Recently, NGS-based transcriptome analyses allow the gene discovery and expression studies in non-model species. Here, we investigated transcriptome profile in order to reveal the key elements that regulate the somatic embryogenesis and to contribute to improve the strategies for *Tilia *in vitro culture. This is the first study reporting transcriptome data in *T. amurensis* and a total of 35,851 unigenes were assembled de novo. We conducted CAP3 assembly following Trinity assembly to merge similar genes, still found that genes have multiple copies as shown in Fig. 6. Provided that *Tilia amurensis* is diploid, it is suggested that the tree species has gene duplications in their genome^14^. Although somatic embryogenesis is usually divided into embryogenic callus and somatic embryo developmental stages in the great majority of studies, we divided it into control (zygotic embryo from seed), SE in globular stage, and developed embryo from isolated SE (Fig. 1). Because, the SE directly emerged from the surface of somatic cell, not going through embryogenic callus stage in *T. amurensis*, which is called ‘direct somatic embryogenesis’. Direct somatic embryogenesis requires profound researches since it has low somaclonal variation rate so that be a desirable approach to obtain somatic embryo identical to parents^15^.

We investigated DEGs to find key genetic factors during somatic embryogenesis. As the result, we identified 1505 DEGs that were considered to be involved in somatic embryo induction and maturation. Then, we performed GO term clustering, network analysis and expression comparison. The series of analyses indicated that transcription regulation, signal transduction and phytohormone signaling were mainly activated during somatic embryogenesis as well as zygotic embryogenesis.

The transcription factors and regulators play important roles in development process^16–18^. In this study, we identified 148 TF/TR genes that were differentially expressed over somatic embryogenesis (Fig. 3). These TF/TR families were associated with functions in embryogenesis and cell differentiation *ZNF, MYB, bHLH, B3* and *b-ZIP*), meristem maintenance or identity (*GRAS* and *NAC*) and hormone signaling (*AP2/ERF* and *AUX/IAA*). *ZNF* family proteins, charging largest portion of TF annotation (Fig. 3), are involved in development processes and differentiation^19^. Among them, *VAL* plays an important role in cell differentiation and is necessary for the development and maintenance of meristems^20^. In the current study, gene *VAL* showed low expression in SE compared to control as in *Quercus suber*^21^. That indicates *VAL* negatively controlled the somatic embryogenesis, which supported by a lot of molecular evidences^22^. In addition, B3 domain containing transcription factors are proven to be involved in embryogenesis and induction of somatic embryo^23,24^. In this study, B3 domain containing genes, such as *LEAFY COTYLEDON2 (LEC2)*, *FUS3* and *ABI3*, generally increased over somatic embryogenesis. The results were consistent with the previous study where *LEC1* and *FUS3* were up-regulated in embryogenic tissue compared to non-embryogenic tissue in *Arabidopsis thaliana*^25^. The result that these TF/TR genes differentially expressed during somatic embryogenesis means the somatic embryogenesis was regulated epigenetically^26^.

The cellular signal transduction associated proteins, such as *LEUCINE-RICH REPEAT CONTAINING RECEPTOR-LIKE KINASE (LRR-RLK)* and *LEA* plays important roles during somatic embryogenesis^27^. For instance, *SOMATIC EMBRYOGENESIS RECEPTOR KINASE (SERK)* has been reported to be expressed specifically during somatic embryogenesis, which allowed it to be a marker for somatic embryogenesis^28,29^. *LdSERK* gene expression level was increased in later stage in European larch^30^, and *DcSERK* was expressed in heart stage of somatic embryo in carrot^28^. In present study, *SERK3* genes were gradually increased through the somatic embryogenesis (Fig. 6, Supplementary Table 1). Moreover, *LEA* genes were considered to be involved in somatic embryogenesis in cotton, white spruce and sweet orange^31–33^. In present study, *LEA* showed high expression in SE stage (Fig. 6, Supplementary Table 1). The results indicate that cellular signal transduction associated genes including *SERK* and *LEA* regulated somatic embryogenesis in *T. amurensis*.

Somatic embryogenesis is controlled by hormonal and metabolic signals^27^. Many genes play the significant roles in somatic embryogenesis actually by regulating plant hormones such as auxin and GA^34^. Auxin biosynthesis and signaling genes such as *YUC, AUX/IAA* and *ARFs* are considered to be required or be an important determinant for somatic embryo induction^9,35^. *YUC* expression were restricted to embryogenic tissues in cotton^36^. Also, *AUX/IAA* genes were actively expressed in the embryogenic callus compared to non-embryogenic callus in cotton and *Catalpa bungei*^37,38^. *ARF* genes were more abundant during the regeneration process in *A. thaliana*^39^, but they are classified as activating and repressing genes^40^. In this study, these auxin related genes were up-regulated during somatic embryogenesis (Figs. 5b and 6, Supplementary Table 1). On the other hand, GA has been reported to decrease somatic embryo induction, although the mechanism that GA regulates somatic embryogenesis is not clear, yet. For example, gibberellin inhibitors improved embryogenic tissue initiation in carrot and conifer^41,42^. Also, the expression of *GIBBERELLIN2-BETA-DIOXYGENASE6 (GA2ox6)*, the GA signaling gene, was negatively correlated to production of somatic embryogenesis in *A. thaliana*^43^. In this study, GA signaling genes were down-regulated during somatic embryogenesis. The results indicate phytohormone auxin and GA signaling genes had the role during somatic embryogenesis. Therefore, auxin and GA signaling genes significantly affected the induction and maturation of somatic embryo in *T. amurensis*.

How genes above influence each other and how the genes regulate somatic embryogenesis can be indirectly inferred through the gene network and expressions of them. First, *PKL* repress the B3 containing genes with the help of the *VAL* proteins^22^. Accordingly, the expressions of *PKL* and *VAL* gene were predicted to be opposite to that of B3 genes. Lower expression of *VAL* and higher expression of B3 genes at SE stage than control suggest that *VAL* genes affected on expression of B3 genes, leading to enhanced somatic embryogenesis. Then, B3 genes are involved in auxin pathway. For instance, *LEC1* was found to up-regulate *YUC10* in *A. thaliana*^44^. And *LEC2* up-regulates *YUC2, YUC4* and *IAA30* in *A. thaliana*^45,46^. In this study, auxin related genes were up-regulated at SE and D stages, where B3 genes showed higher expression compared to control (Fig. 6). Various expression patterns of auxin signaling genes during somatic embryogenesis might arise from the different role of the genes; *YUCs* are involved in biosynthesis and *AUX/IAA* and *ARF* in signaling of auxin. Otherwise, that might be related to repression of *ARF* genes by *AUX/IAA* genes in auxin signaling pathway. Besides, *LEA* genes co-express with *IAA* genes, although it does not suggest regulatory relationship (Fig. 5b)^47^. Meanwhile, The B3 domain containing genes are involved in GA signaling^45^. For instance, *LEC2* and *FUS3* repress expression of *GIBBERELLIN3-BETA-DIOXYGENASE2 (GA3ox2)* and *GA3ox1*^48,49^. Gibberellin oxidases, which catabolize biologically active GA, were down-regulated during somatic embryogenesis in this study (Fig. 6). This indicates gibberellin oxidases were negatively regulated by B3 genes for induction of somatic embryo.

The up- and down-regulated genes used in Fig. 7 were selected as having a large difference in RNA-Seq TMM value by stage (|z-score|> 2.3 for SE stage). The genes included the genes related to development such as *EXTENSIN*, late embryogenesis related gene and transcription factors as well as genes that seems not to be relevant (Supplementary Table 2). Some of the genes were either uncharacterized genes or a gene with previously unreported sequence. Although experimental proof is required, the gene might play a role in somatic embryogenesis so that we provide that new sequence (Supplementary data 1).

## Materials and methods

### Plant materials

Somatic embryos were induced from the immature zygotic embryos of *T. amurensis* which were collected from a tree of clonal seed orchard (established by Korea Forest Seed & Variety Center and located in Hwasung-si, South Korea) in August 2019. Seeds were sterilized in 70% (v/v) ethanol for 1 min followed by disinfecting in 2% (v/v) sodium hypochlorite solution for 8 min, and were rinsed 5 times in sterile distilled water at clean-bench. Somatic embryogenesis procedure was accomplished on MS (Murashige and Skoog)^50^ media with 2,4-D 1.0 mg/L following previous reports^12^. Somatic embryogenesis was induced in the dark room which is controlled temperature in 25 ± 2 ℃ and humidity 40%. After somatic embryos were induced, they were separated from explants and cultured on MS media without any plant regulator. All media were adjusted to pH 5.8 and sterilized for 15 min at 120 ℃. Media were solidified with gelrite 0.3% (w/v) on petri plates (9 cm in diameter).

### RNA extraction and sequencing

Total RNA was extracted from C, SE and D using the HiYield Total RNA Mini Kit (Plant) following manufacturer’s instruction. The purity of each RNA sample was assessed by Thermo Scientific NanoDrop and Agilent 4200 TapeStation. Library preparation and sequencing were performed using Illumina HiSeq by Macrogen (Seoul, Korea).

### Bioinformatic analysis of sequence data

Raw RNA-Seq reads were filtered and trimmed for low-quality regions using PRINSEQ-lite (v0.20.4). Then, with clean reads, de novo assembly of *Tilia amurensis* embryo transcriptome was conducted using Trinity (v2.8.5). For delicate assembly, annotation using TransDecoder and merging unigenes with CAP3 were followed^51^. Then genome coverage of assembled unigenes were tested by Benchmarking Universal Single-Copy Orthologs (BUSCO). BLAST search against both Plant Refseq protein and GenBank non-redundant protein sequences were carried out to assign the putative functions to assembled genes using BLASTx program (v2.10.0)^52^. To identify putative transcription factors and regulators, iTAK online (v1.6) was used^53^. Reads for each sample were counted using method RSEM^54^ and DEGs were identified using edgeR^55^ with the cut-off *p*-value < 1e^−5^ and log_2_FC > 2. Functional annotation by gene ontology (GO) and KEGG^56–58^ was analyzed using DAVID (v6.8)^59^. Gene functional interaction network was constructed using Cytoscape GeneMANIA app using *A. thaliana* genes which correspond to DEGs^60^.

### Quantitative real-time PCR validation

Gene-specific primers (Supplementary Table 3) were designed for the cDNAs sequences with Primer3 (v0.4.0, http://bioinfo.ut.ee/primer3-0.4.0/) and synthesized commercially. RNA samples were adjusted to concentration of 30 ng/ul and first-strand cDNA was generated using TOPscript RT DryMIX (Enzynomics). Then qRT-PCR was performed on a Bio-Rad CFX96, using the TOPreal qPCR 2X PreMIX (Enzynomics). PCR was carried out as follows: initial denaturing at 95 ℃ for 10 min, 40cycles consisting of 95 ℃ for 10 s, 55 ℃ for 15 s, and 72 ℃ for 15 s. The reference gene for normalization was ubiquitin gene showing stable expression. The experiments were carried out with three technical repetitions for each sample. Pearson’s correlation coefficient of log-scaled qPCR and RNA-Seq values were calculated using R (v3.6.2). All methods comply with local, national or international guidelines and legislation for this study.

## Conclusions

We could reveal the molecular mechanism of somatic embryogenesis by analyzing the transcriptomes and comparing the expression levels. Somatic embryogenesis was triggered by the process that transcription factors, interacting with signaling transduction genes, regulated phytohormones in *Tilia amurensis.* Since there have been only few studies about direct somatic embryogenesis, this study would provide valuable insights of molecular mechanism for direct somatic embryogenesis in woody plant.

## Supplementary Information

Supplementary Information 1.

Supplementary Information 2.

Supplementary Information 3.
