# Supplementary material for: Comparative transcriptome analysis during developmental stages of direct somatic embryogenesis in Tilia amurensis Rupr
Source: Sci Rep. 2021 Mar 18;11:6359. doi: 10.1038/s41598-021-85886-z (PMC7973583; doi:10.1038/s41598-021-85886-z)
Supplement: Supplementary file 1 — Supplementary Information 1. [file 41598_2021_85886_MOESM1_ESM.docx]

Comparative transcriptome analysis during developmental stages of direct somatic embryogenesis in *Tilia amurensis* Rupr.

Hye-In Kang^1, 2^, Chae-Bin Lee^2^, Soon-Ho Kwon^1^, Ji-Min Park^2^, Kyu-Suk Kang^2,^* and Donghwan Shim^1,3,^*

^1^ Department of Forest Bio-Resources, National Institute of Forest Science, Suwon, 13361, Republic of Korea

^2^ Department of Agriculture, Forestry and Bioresources, College of Agriculture and Life Sciences, Seoul National University, Seoul, 08826, Republic of Korea

^3^ Department of Biological Sciences, Chungnam National University, Daejeon, 34134, Republic of Korea

***** Correspondence: KS Kang, [kangks84@snu.ac.kr](mailto:kangks84@snu.ac.kr); D Shim, shim.donghwan@gmail.com

**Supplementary data 1. Sequence of LOC24451**

**>LOC24451**

**TTTTTTTTTAAAAAAAAAAAGGCATGCATAACAGGAGATTGATTCATCAGATAAAATTAA**

**AAGTGCAATACAACCATAGTTCAATTACAAAGCAGAAAGATATGCTTTAAACTGTTCCAC**

**AAACCTAATATAACTCTATAAAATACAAAGAAAGCAAAGGGGAAAAAGGGTGATCGAGGA**

**ACCACGCGAAAATTAAGCCTTGAACTGCTCCACAGAAACCAACGGAGCTAACTGCCATAT**

**AATCAATCATAAGATAAAATATCTATATTAATTCAAGAAACAAGAAATTCATCGGGGAAA**

**TCTACCTGAAAGATTGGGAAATGTAATCAGTCGCCAATATTTAGTTTAAGCAAAGCCGCG**

**GTTAGTTGAGCATCCGGCGAGTCAGCTGGCAACAGAGGACGAATCACGTCCCTCATTTGG**

**GTGATTGTCAGGTAATGCTCTTCCAGGGCTCTTAAAGTGCTGGTGGTCTCTGCCTTCATA**

**TTTGCTAATGTTGCTTTCAGCCTTTTCACTTTCTTCTCCACTTTCTTGACCCTCTTCCAT**

**GAATTATTCTTGCTTGCTTTCCTTTCATTTCTTGGGCCCATAATATTTTTCTCTTTTTTC**

**AGATAGAGAGAGCGCTGTGGGAAGTGAGGAGAGATGAAGTGAAG**
