# Supplementary material for: Comparative transcriptome analysis during developmental stages of direct somatic embryogenesis in Tilia amurensis Rupr
Source: Sci Rep. 2021 Mar 18;11:6359. doi: 10.1038/s41598-021-85886-z (PMC7973583; doi:10.1038/s41598-021-85886-z)
Supplement: Supplementary file 2 — Supplementary Information 2. [file 41598_2021_85886_MOESM2_ESM.docx]

Comparative transcriptome analysis during developmental stages of direct somatic embryogenesis in *Tilia amurensis* Rupr.

Hye-In Kang^1, 2^, Chae-Bin Lee^2^, Soon-Ho Kwon^1^, Ji-Min Park^2^, Kyu-Suk Kang^2,^* and Donghwan Shim^1,3,^*

^1^ Department of Forest Bio-Resources, National Institute of Forest Science, Suwon, 13361, Republic of Korea

^2^ Department of Agriculture, Forestry and Bioresources, College of Agriculture and Life Sciences, Seoul National University, Seoul, 08826, Republic of Korea

^3^ Department of Biological Sciences, Chungnam National University, Daejeon, 34134, Republic of Korea

***** Correspondence: KS Kang, [kangks84@snu.ac.kr](mailto:kangks84@snu.ac.kr); D Shim, shim.donghwan@gmail.com


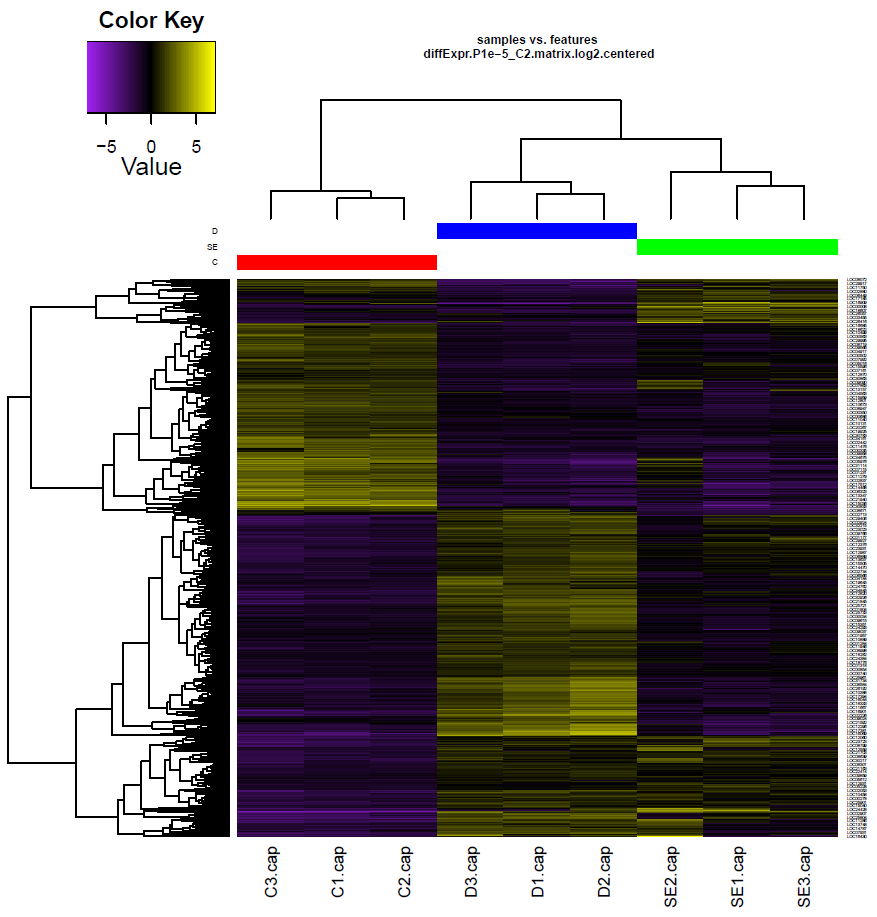


**Supplementary Fig 1.** Correlation heatmap of samples to genes. Expression values are log2-transformed median-centered TMM-normalized TPM. Color Key indicates Row Z-scores of expression values.


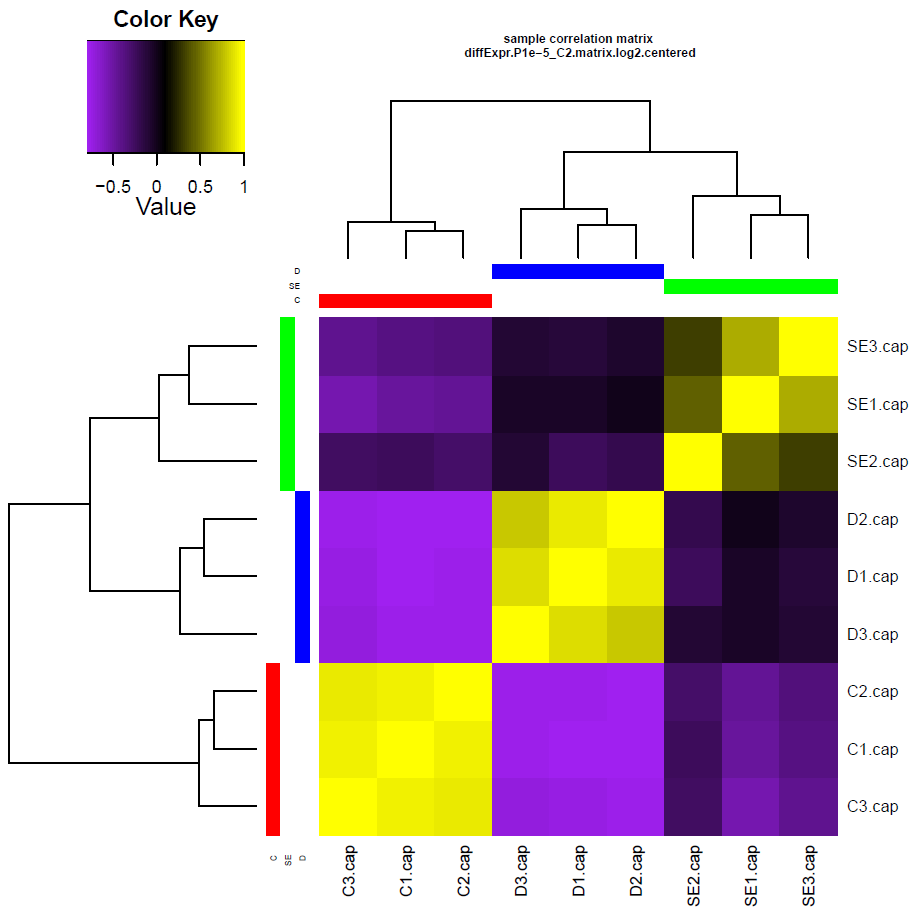

**Supplementary Fig 2.** Sample correlation heatmap. Clustered heatmap showing the Pearson correlation matrix for pairwise sample comparisons.


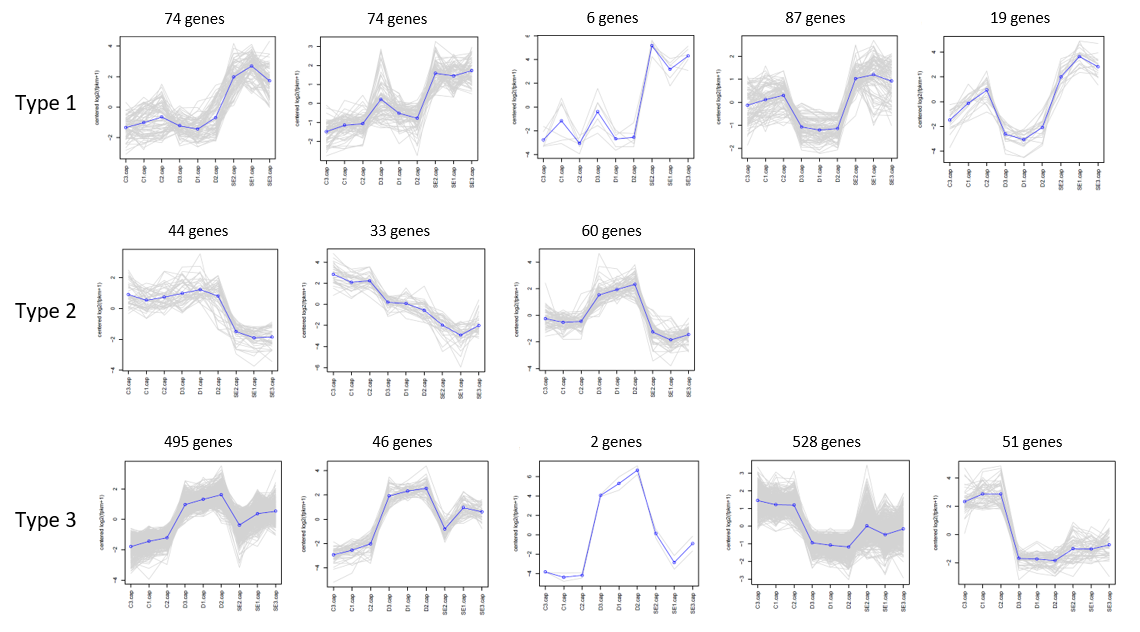


**Supplementary Fig 3.** Clustering of differentially expressed genes during somatic embryogenesis in *T. amurensis* based on their expression modulation. Type 1, up-regulated genes in SE; Type 2, down-regulated in SE; Type 3, up- or down-regulated during somatic embryogenesis.


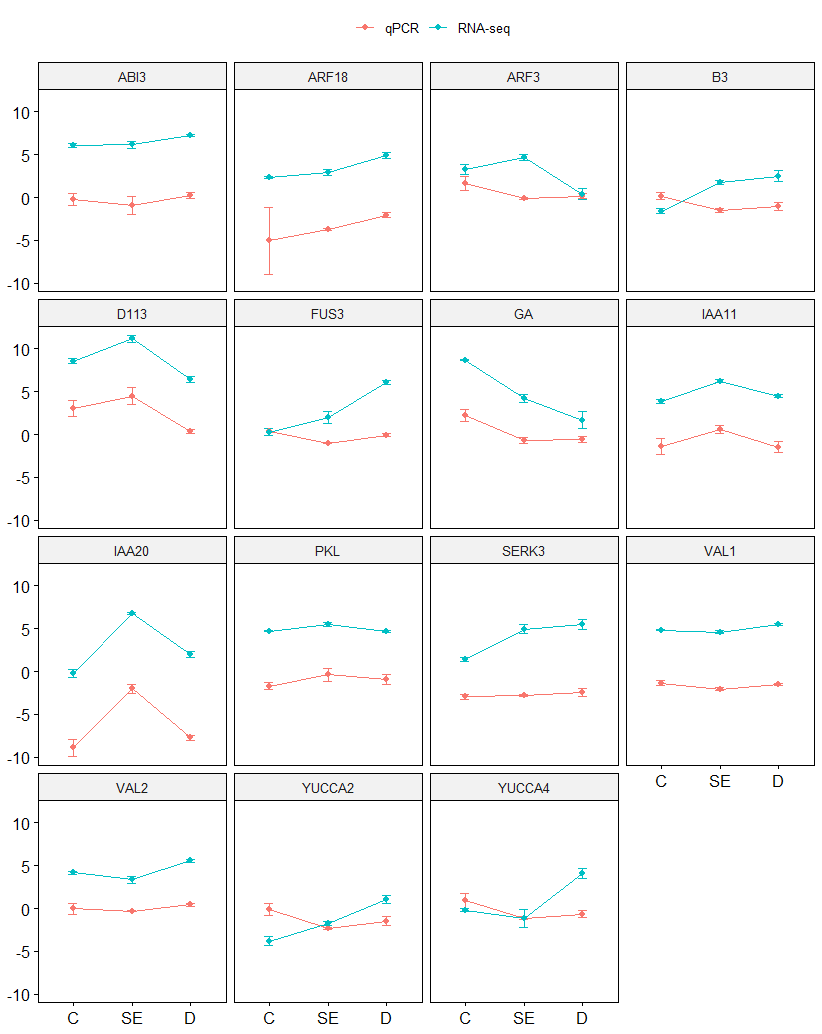


(continued)


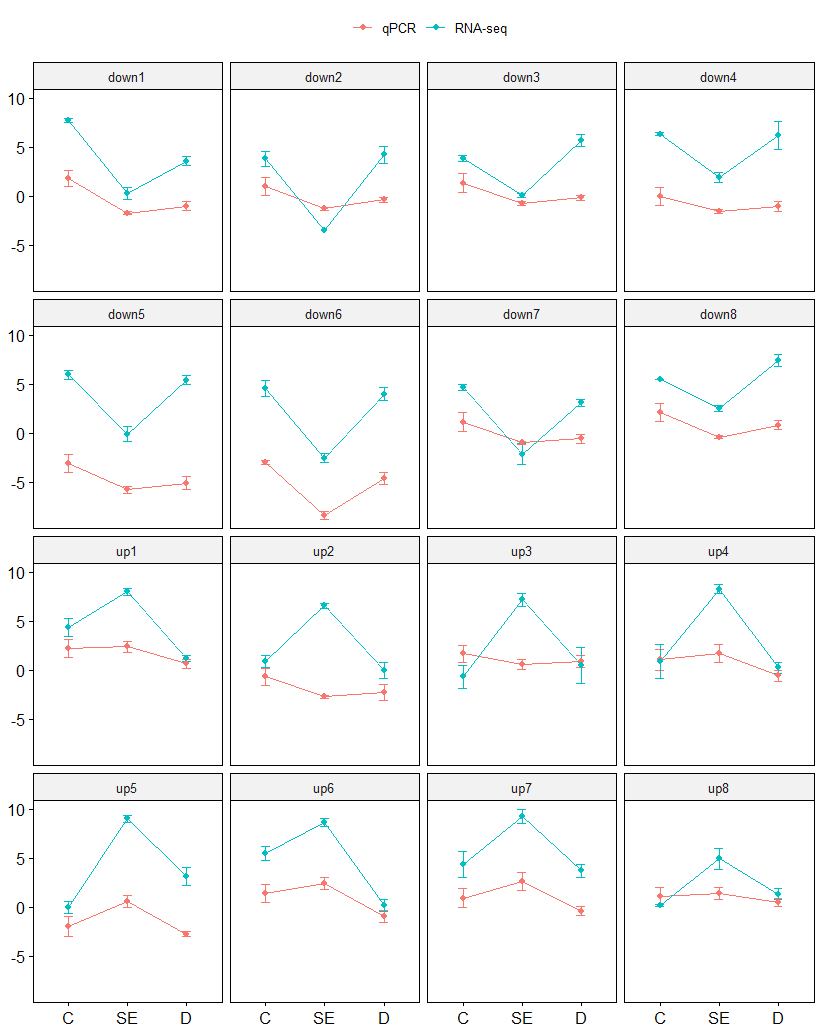


**Supplementary Fig 4.** Comparing the expression of each genes in the RNA-seq dataset and by qRT-PCR. Blue and red lines represent the change of log_2_(TMM) and log_2_(qPCR ΔCT) values. Mean ± sd.
