# Supplementary material for: Comparative transcriptome analysis during developmental stages of direct somatic embryogenesis in Tilia amurensis Rupr
Source: Sci Rep. 2021 Mar 18;11:6359. doi: 10.1038/s41598-021-85886-z (PMC7973583; doi:10.1038/s41598-021-85886-z)
Supplement: Supplementary file 3 — Supplementary Information 3. [file 41598_2021_85886_MOESM3_ESM.docx]

Comparative transcriptome analysis during developmental stages of direct somatic embryogenesis in *Tilia amurensis* Rupr.

Hye-In Kang^1, 2^, Chae-Bin Lee^2^, Soon-Ho Kwon^1^, Ji-Min Park^2^, Kyu-Suk Kang^2,^* and Donghwan Shim^1,3,^*

^1^ Department of Forest Bio-Resources, National Institute of Forest Science, Suwon, 13361, Republic of Korea

^2^ Department of Agriculture, Forestry and Bioresources, College of Agriculture and Life Sciences, Seoul National University, Seoul, 08826, Republic of Korea

^3^ Department of Biological Sciences, Chungnam National University, Daejeon, 34134, Republic of Korea

***** Correspondence: KS Kang, [kangks84@snu.ac.kr](mailto:kangks84@snu.ac.kr); D Shim, shim.donghwan@gmail.com

**Supplementary Table 1.** Highly expressed genes in each group

| Group | Gene ID | BLAST | | |
| --- | --- | --- | --- | --- |
|  |  | Accession no. | Description | Species |
| C-high group | LOC01157 | XP_007009698.2 | PREDICTED: ethylene-responsive transcription factor RAP2-7 isoform X2 | *Theobroma cacao* |
|  | LOC02221 | XP_007012724.1 | PREDICTED: myosin-17 | *Theobroma cacao* |
|  | LOC02462 | XP_007031605.2 | PREDICTED: NAC domain-containing protein 8 isoform X2 | *Theobroma cacao* |
|  | LOC03174 | XP_017977121.1 | PREDICTED: actin | *Theobroma cacao* |
|  | LOC04033 | XP_007009294.2 | PREDICTED: wall-associated receptor kinase-like 20 | *Theobroma cacao* |
|  | LOC04493 | XP_007015149.2 | PREDICTED: hexokinase-3 | *Theobroma cacao* |
|  | LOC06143 | XP_017982700.1 | PREDICTED: glucan endo-1,3-beta-glucosidase 14 | *Theobroma cacao* |
|  | LOC08266 | XP_007012309.2 | PREDICTED: heat shock factor protein HSF30 | *Theobroma cacao* |
|  | LOC09106 | XP_007047716.2 | PREDICTED: L-type lectin-domain containing receptor kinase IX.1 | *Theobroma cacao* |
|  | LOC09988 | XP_017972249.1 | PREDICTED: ethylene-responsive transcription factor CRF4 | *Theobroma cacao* |
|  | LOC10142 | XP_017977979.1 | PREDICTED: CBL-interacting serine/threonine-protein kinase 25 | *Theobroma cacao* |
|  | LOC10146 | XP_017979113.1 | PREDICTED: pentatricopeptide repeat-containing protein At4g16470 isoform X2 | *Theobroma cacao* |
|  | LOC10612 | XP_007011728.2 | PREDICTED: CBL-interacting serine/threonine-protein kinase 25 | *Theobroma cacao* |
|  | LOC12975 | XP_017981424.1 | PREDICTED: uncharacterized protein LOC18592011 isoform X1 | *Theobroma cacao* |
|  | LOC13928 | XP_007043870.2 | PREDICTED: NAC domain-containing protein 83 | *Theobroma cacao* |
|  | LOC15081 | XP_007015149.2 | PREDICTED: hexokinase-3 | *Theobroma cacao* |
|  | LOC16331 | XP_017982169.1 | PREDICTED: F-box protein PP2-B11 | *Theobroma cacao* |
|  | LOC18254 | XP_017977600.1 | PREDICTED: DNA-directed RNA polymerase III subunit RPC8 isoform X2 | *Theobroma cacao* |
|  | LOC19018 | XP_007017989.1 | PREDICTED: probable serine/threonine-protein kinase At4g35230 | *Theobroma cacao* |
|  | LOC19289 | XP_017981783.1 | PREDICTED: ubiquitin-conjugating enzyme E2-17 kDa isoform X1 | *Theobroma cacao* |
|  | LOC20644 | XP_007018006.2 | PREDICTED: pre-rRNA-processing protein TSR1 homolog | *Theobroma cacao* |
|  | LOC20765 | XP_017980375.1 | PREDICTED: heat stress transcription factor A-4c isoform X2 | *Theobroma cacao* |
|  | LOC31460 | XP_017979180.1 | PREDICTED: LOW QUALITY PROTEIN: plastidic ATP/ADP-transporter | *Theobroma cacao* |
|  | LOC32668 | XP_007039732.2 | PREDICTED: ethylene-responsive transcription factor 2 | *Theobroma cacao* |
| SE-high group | LOC07646 | XP_007046353.2 | PREDICTED: ABC transporter G family member 21 | *Theobroma cacao* |
|  | LOC13025 | XP_007050158.2 | PREDICTED: auxin-responsive protein IAA11 | *Theobroma cacao* |
|  | LOC13910 | XP_017970733.1 | PREDICTED: ABC transporter C family member 12 isoform X2 | *Theobroma cacao* |
|  | LOC14434 | XP_007013516.2 | PREDICTED: auxin-responsive protein IAA20 | *Theobroma cacao* |
|  | LOC15176 | XP_017982252.1 | PREDICTED: probable LRR receptor-like serine/threonine-protein kinase At1g56130 | *Theobroma cacao* |
|  | LOC16132 | XP_007028984.2 | PREDICTED: late embryogenesis abundant protein D-113 | *Theobroma cacao* |
|  | LOC22799 | XP_017972709.1 | PREDICTED: uncharacterized protein LOC18606060 isoform X2 | *Theobroma cacao* |
|  | LOC24895 | XP_007021328.2 | PREDICTED: NAC domain-containing protein 72 | *Theobroma cacao* |
|  | LOC25998 | XP_017970733.1 | PREDICTED: ABC transporter C family member 12 isoform X2 | *Theobroma cacao* |
| D-high group | LOC02010 | XP_017976763.1 | PREDICTED: cyclin-D3-3 | *Theobroma cacao* |
|  | LOC02048 | XP_017975859.1 | PREDICTED: kinesin-like protein KIN12B | *Theobroma cacao* |
|  | LOC02183 | XP_017978080.1 | PREDICTED: cyclin-A2-4 | *Theobroma cacao* |
|  | LOC03974 | XP_007050754.2 | PREDICTED: rho GTPase-activating protein 2 | *Theobroma cacao* |
|  | LOC04321 | XP_017975859.1 | PREDICTED: kinesin-like protein KIN12B | *Theobroma cacao* |
|  | LOC07106 | XP_017982431.1 | PREDICTED: methyl-CpG-binding domain-containing protein 7 | *Theobroma cacao* |
| Gradually increased group | LOC00353 | XP_017975298.1 | PREDICTED: ABC transporter B family member 19 isoform X1 | *Theobroma cacao* |
|  | LOC00434 | XP_007038631.2 | PREDICTED: leucine-rich repeat receptor protein kinase EMS1 | *Theobroma cacao* |
|  | LOC00491 | XP_017974988.1 | PREDICTED: probable inactive leucine-rich repeat receptor-like protein kinase At3g03770 isoform X1 | *Theobroma cacao* |
|  | LOC00562 | XP_017979204.1 | PREDICTED: homeobox-leucine zipper protein ATHB-14 isoform X1 | *Theobroma cacao* |
|  | LOC00681 | XP_017977929.1 | PREDICTED: leucine-rich repeat receptor-like tyrosine-protein kinase PXC3 isoform X1 | *Theobroma cacao* |
|  | LOC00706 | XP_017975800.1 | PREDICTED: leucine-rich repeat receptor-like protein kinase PXC2 | *Theobroma cacao* |
|  | LOC01302 | XP_017978856.1 | PREDICTED: inactive protein kinase SELMODRAFT_444075 | *Theobroma cacao* |
|  | LOC01422 | XP_007034748.2 | PREDICTED: probable serine/threonine-protein kinase WNK9 | *Theobroma cacao* |
|  | LOC01573 | XP_017981462.1 | PREDICTED: cyclin-D3-1 isoform X2 | *Theobroma cacao* |
|  | LOC01656 | XP_007023449.1 | PREDICTED: putative receptor-like protein kinase At1g80870 | *Theobroma cacao* |
|  | LOC01802 | XP_007042447.2 | PREDICTED: probable receptor-like protein kinase At1g49730 isoform X1 | *Theobroma cacao* |
|  | LOC02131 | XP_017978889.1 | PREDICTED: auxin response factor 3 isoform X4 | *Theobroma cacao* |
|  | LOC02277 | XP_017969715.1 | PREDICTED: growth-regulating factor 1 isoform X1 | *Theobroma cacao* |
|  | LOC02789 | XP_007012668.2 | PREDICTED: growth-regulating factor 1 isoform X1 | *Theobroma cacao* |
|  | LOC03438 | XP_007021090.2 | PREDICTED: kinesin-3 | *Theobroma cacao* |
|  | LOC04411 | XP_007038447.2 | PREDICTED: BRASSINOSTEROID INSENSITIVE 1-associated receptor kinase 1 | *Theobroma cacao* |
|  | LOC04874 | XP_007038447.2 | PREDICTED: BRASSINOSTEROID INSENSITIVE 1-associated receptor kinase 1 | *Theobroma cacao* |
|  | LOC06465 | XP_007045573.2 | PREDICTED: inactive leucine-rich repeat receptor-like serine/threonine-protein kinase At1g60630 | *Theobroma cacao* |
|  | LOC06581 | XP_007010597.2 | PREDICTED: probable inactive leucine-rich repeat receptor-like protein kinase At3g03770 | *Theobroma cacao* |
|  | LOC07309 | XP_007017159.2 | PREDICTED: probable inactive receptor kinase At5g67200 | *Theobroma cacao* |
|  | LOC09238 | XP_017973802.1 | PREDICTED: LRR receptor-like serine/threonine-protein kinase FEI 2 isoform X1 | *Theobroma cacao* |
|  | LOC09600 | XP_007024621.2 | PREDICTED: growth-regulating factor 5 | *Theobroma cacao* |
|  | LOC10150 | XP_017975012.1 | PREDICTED: glutamine synthetase leaf isozyme, chloroplastic | *Theobroma cacao* |
|  | LOC11124 | XP_017983375.1 | PREDICTED: uncharacterized protein LOC18587787 isoform X1 | *Theobroma cacao* |
|  | LOC11361 | XP_007051458.2 | PREDICTED: rho GTPase-activating protein 3 | *Theobroma cacao* |
|  | LOC11393 | XP_007050516.2 | PREDICTED: VAN3-binding protein | *Theobroma cacao* |
|  | LOC12192 | XP_007014619.2 | PREDICTED: probable receptor-like protein kinase At5g56460 | *Theobroma cacao* |
|  | LOC12705 | XP_017977751.1 | PREDICTED: leucine-rich repeat receptor-like protein kinase PXC1 | *Theobroma cacao* |
|  | LOC12815 | XP_007042498.2 | PREDICTED: cyclin-D3-1 | *Theobroma cacao* |
|  | LOC13231 | XP_007018432.2 | PREDICTED: growth-regulating factor 1 | *Theobroma cacao* |
|  | LOC16097 | XP_017972194.1 | PREDICTED: putative B3 domain-containing protein At5g66980 | *Theobroma cacao* |
|  | LOC17162 | XP_007019986.2 | PREDICTED: 50S ribosomal protein L21, chloroplastic | *Theobroma cacao* |
|  | LOC21682 | XP_007038447.2 | PREDICTED: BRASSINOSTEROID INSENSITIVE 1-associated receptor kinase 1 | *Theobroma cacao* |
|  | LOC25935 | XP_017974082.1 | PREDICTED: ABC transporter G family member 5 | *Theobroma cacao* |
|  | LOC26238 | XP_007050516.2 | PREDICTED: VAN3-binding protein | *Theobroma cacao* |
|  | LOC27236 | XP_017983952.1 | PREDICTED: homeobox protein ATH1 | *Theobroma cacao* |

**Supplementary Table 2.** Genes that up- and down-regulated in SE and their expression (Z-normalized TMM) and BLAST results.

| Gene | Index | Z-score | | | BLAST accession no. | Gene description |
| --- | --- | --- | --- | --- | --- | --- |
|  |  | C | SE | D |  |  |
| up1 | LOC14731 | -0.27896 | 3.285258 | -3.0063 | XP_017978735.1 | PREDICTED: uncharacterized protein LOC108662584 |
| up2 | LOC16039 | -1.52033 | 3.497572 | -1.97724 | XP_007051044.2 | PREDICTED: uncharacterized protein LOC18613646 |
| up3 | LOC20816 | -2.38647 | 3.927179 | -1.54071 | XP_016726747.1 | PREDICTED: 21 kDa seed protein-like |
| up4 | LOC23299 | -1.7517 | 4.620733 | -2.86904 | XP_022728671.1 | extensin-like |
| up5 | LOC30816 | -3.42789 | 4.541692 | -1.11381 | XP_006847692.1 | beta-glucosidase 13 isoform X2 |
| up6 | LOC08765 | 0.430451 | 3.547963 | -3.97841 | XP_022737370.1 | low-temperature-induced 65 kDa protein-like |
| up7 | LOC14323 | -1.35621 | 3.375057 | -2.01884 | XP_016714241.1 | PREDICTED: late embryogenesis abundant protein D-34 |
| up8 | LOC23898 | -1.54915 | 2.343747 | -0.7946 | XP_021649608.1 | uncharacterized protein LOC110641995 |
| down1 | LOC03223 | 3.667759 | -3.28066 | -0.3871 | XP_022727198.1 | transcription factor bHLH162-like |
| down2 | LOC09310 | 1.156135 | -2.71558 | 1.559447 | XP_022729885.1 | probable LRR receptor-like serine/threonine-protein kinase At1g05700 |
| down3 | LOC12819 | 0.36077 | -2.50378 | 2.14301 | XP_022773430.1 | hydroquinone glucosyltransferase-like |
| down4 | LOC18136 | 1.397942 | -2.67667 | 1.278731 | XP_007029839.2 | PREDICTED: oxygen-evolving enhancer protein 2, chloroplastic |
| down5 | LOC24451 | 1.833397 | -3.11984 | 1.286446 | - | - |
| down6 | LOC12770 | 1.651009 | -2.7416 | 1.090588 | XP_016708967.1 | PREDICTED: protein PLANT CADMIUM RESISTANCE 7-like |
| down7 | LOC12530 | 1.896079 | -2.3792 | 0.483121 | XP_022717671.1 | probable F-box protein At4g22030 |
| down8 | LOC17954 | 0.25963 | -2.46427 | 2.204644 | XP_021906846.1 | histone H2AX-like |

**Supplementary Table 3.** Primers for qPCR validation

| Gene | Gene ID | Forward (5’ -> 3’) | Reverse(5’ -> 3’) |
| --- | --- | --- | --- |
| ubiquitin | LOC11837 | AGGGCAATCAACCATGCTAC | CAGCCCCAACATGAGAAGTT |
| PKL | LOC04658 | CCAAAAATTCTTCCCGGTTT | ATGAGCTCTTGCCATTGCTT |
| VAL1 | LOC00762 | TGAAGATCCAGTGCAGCAAC | TGGACGAAGCAAGTCTTGTG |
| VAL2 | LOC01205 | GGAATTCAGCTGGTGGTGAT | AGCAACCTCTTGCTTTTGGA |
| SERK3 | LOC04411 | CGCTTCCCAGAAAGAACTTG | TGTCTTCGCAACACTCAAGG |
| B3 | LOC16097 | GGCATACCACCTGCTTTTGT | AATAGTCACGCGCCTTTCAC |
| FUS3 | LOC16901 | TAACACGTGCTGGGAGAGTG | GTTGACCCGTGACCTTGTCT |
| ABI3 | LOC00760 | TTCTGGGAGATGGGTTTCTG | CAGGGATGGAAACCTGAGAA |
| GA2ox1 | LOC12104 | TCCATTGCCATCTCTGATGA | TGCATGAGCCTGTTATCTGC |
| D-113 | Loc16132 | GCTCCCAGTCGTCGAATAAG | GGTACAACAGAGGCGATGCT |
| YUCCA4 | LOC02594 | AGTACCCAACCAAGCACCAG | ATGATCGAACTCTGCGCTTT |
| YUCCA2 | LOC09060 | AAAGGCTGTTTTGGTGGTTG | AAGACGTGCACCGAGTCTCT |
| IAA20 | LOC03872 | CGATGTTCCTTGGGAGATGT | CTGGTTTTCACTCCCCAAAA |
| IAA11 | LOC13025 | GAGGGTGACTGGATGCTTGT | GATGGGCTTGCTTCTTTGTC |
| ARF18 | LOC27068 | TTCAGGTGTGCCATTTTTGA | AGACGAAGGCATTGTGCTCT |
| ARF3 | LOC17199 | TCCAATCGGAAACGATAACC | AAGGGTGCCATGGAATAGTG |
| up1 | LOC14731 | CAACGAGCGTTCCTTTAACC | CAAGTGGGACCTTCACCAGT |
| up2 | LOC16039 | GGCGTAGAAAAGCGACTGAG | AGATGCCTCTGGCTCAAAGA |
| up3 | LOC20816 | TGGGTCTGATTGGAAGGAAC | CTTGTCCGGAAATCGTTGTT |
| up4 | LOC23299 | TCCACCTCCCACTCAAGTTC | AGTTTCGGAAGGTGGAGGAT |
| up5 | LOC30816 | CGGAGGAGACCTCTGTTGAG | GATCAGAGACGGTTGGTGGT |
| up6 | LOC08765 | ATGAACACCCATGATGAGCA | GCAATTGGTTTGGGAGAGAA |
| up7 | LOC14323 | GGTTTCAGCTGTCTGCATCA | GCAGCTCAACGTGAGAATCA |
| up8 | LOC23898 | TTTCAATCATGCCAGCAAAG | CCATGGGATCCTCAAAGAGA |
| down1 | LOC03223 | GGGTGTGAATAAGGGAACGA | TGAGCCCGGAGTACAAATTC |
| down2 | LOC09310 | GTGACAGGCAACCCAGATTT | TGGTGCCTTGCTCTTTTTCT |
| down3 | LOC12819 | GACTGATTGAAGGGGACGAA | CATTGGCCAGTGATTTTGTG |
| down4 | LOC18136 | TGCACTTGACCAAAAACCAA | GTGTGTGGATGATGGCAGAG |
| down5 | LOC24451 | GAGGAACCACGCGAAAATTA | TTTCCCCGATGAATTTCTTG |
| down6 | LOC12770 | CGCTGAAATTGTGGATGATG | TTCGGTACGTGCATGACATT |
| down7 | LOC12530 | TTGGAGGAAGGAGAAGCTGA | TGCATCATCTGCAAGGTCTC |
| down8 | LOC17954 | GTCCCAAGGCATGTTCAGTT | CCTTCTTTGGCAGCAGATTC |
